# The medium, the message and the measure: a theory-driven review on the value of telehealth as a patient-facing digital health innovation

**DOI:** 10.1186/s13561-019-0239-5

**Published:** 2019-07-03

**Authors:** Seye Abimbola, Sarah Keelan, Michael Everett, Kim Casburn, Michelle Mitchell, Katherine Burchfield, Alexandra Martiniuk

**Affiliations:** 1 School of Public Health, University of Sydney, Sydney, Australia; 2 Royal Far West, Sydney, Australia; 3 The George Institute for Global Health, Sydney, Australia; 4 University of Toronto, Toronto, Canada

**Keywords:** Telehealth, Digital health, Value of information, Utility, Transaction costs, Satisfaction, Innovation

## Abstract

By what measure should a policy maker choose between two mediums that deliver the same or similar message or service? Between, say, video consultation or a remote patient monitoring application (i.e. patient-facing digital health innovations) and in-person consultation? To answer this question, we sought to identify measures which are used in randomised controlled trials. But first we used two theories to frame the effects of patient-facing digital health innovations on – 1) transaction costs (i.e. the effort, time and costs required to complete a clinical interaction); and 2) process outcomes and clinical outcomes along the care cascade or information value chain, such that the ‘value of information’ (VoI) is different at each point in the care cascade or value chain. From the trials, we identified three categories of measures: outcome (process or clinical), satisfaction, and cost. We found that although patient-facing digital health innovations tend to confer much of their value by altering process outcomes, satisfaction, and transaction costs, these measures are inconsistently assessed. Efforts to determine the relative value of and choose between mediums of service delivery should adopt a metric (i.e. mathematical combination of measures) that capture all dimensions of value. We argue that ‘value of information’ (VoI) is such a metric – it is calculated as the difference between the ‘expected utility’ (EU) of alternative options. But for patient-facing digital health innovations, ‘expected utility’ (EU) should incorporate the probability of achieving not only a clinical outcome, but also process outcomes (depending on the innovation under consideration); and the measures of utility should include satisfaction and transaction costs; and also changes in population access to services, and health system capacity to deliver more services, which may result from reduction in transaction costs.

## Introduction

The primary value of patient-facing digital health innovations (e.g. video consultations, text message reminders and care navigation support) may not be their effects on clinical outcomes [1–4]. Delivering the same service through a different medium may only result in improved process outcomes for the patient or provider. Any improvements in clinical outcomes would probably occur due to improved process of care, or because patients and providers are able to do things through the medium that are not feasible in in-person interaction e.g. the possibility of increased frequency of monitoring of patients with chronic disease [2–5]. Hence, it is important that *the measure(s)* used to evaluate these patient-facing digital health innovations can separate process outcomes from clinical outcomes, and when mediums are substitutable, can also separate the effects of *the medium* from *the message*.

Having such a *measure* may be important for making the case to regulators or third party payers to pay for or subsidise the costs of using patient-facing digital health innovations [6]. Notably, the need for such a measure is borne out in our experience working in a non-governmental organisation which seeks insurance reimbursement for services provided via videoconferencing to children in rural Australia [7]. Such a measure is important in efforts to assess the value proposition of health technologies, as the value or relative advantage that a technology confers may be different for diverse stakeholders [8–11]. The development of health technologies has been characterized by poor alignment between supply-side and demand-side value [9]. This poor alignment may manifest in the willingness (or lack thereof) of stakeholders to pay or share the costs of such technologies [9–11].

Our challenge was therefore to identify existing evidence that demonstrates the real and potential value of videoconferencing. However we identified from cursory literature searches that randomised controlled trials evaluating the effects of videoconferencing and other patient-facing digital health innovations did not typically disentangle the effects of the medium from the message – e.g. studies often compared groups in which patients received a service remotely, with another group (often usual care) in which the patient did not receive the same service. These studies provide evidence for the impact of only the service and not the impact of the medium of service delivery, or the impact of the service combined with the impact of the medium of delivery. We were interested in studies that separated the effects of the message from the medium – allowing us the possibility of identifying benefits or harms (process or clinical) that may be attributed to the medium of delivery.

Our aim in this paper was to address the question we encountered in practice: *by what measure should a policy maker choose between two mediums that deliver the same or similar message or service?* That is, how do we make the policy argument to a regulator or third-party payer that an alternative medium of service delivery (i.e. videoconferencing) which delivers the same “message” that could be delivered in-person is worth paying for or subsidising? We therefore identified existing randomised controlled trials which could distinguish the relative value of one medium compared to another in delivering the same message or service; and the measures used in each to trial to assess process outcomes separate from clinical outcomes. Our analysis was informed by framing the essential role of the patient-facing use of telehealth as facilitating patient-provider interactions – by bringing service providers and users together more seamlessly; and by reducing frictions in the interaction between parties to a transaction or information exchange.

**Fig. 1:**
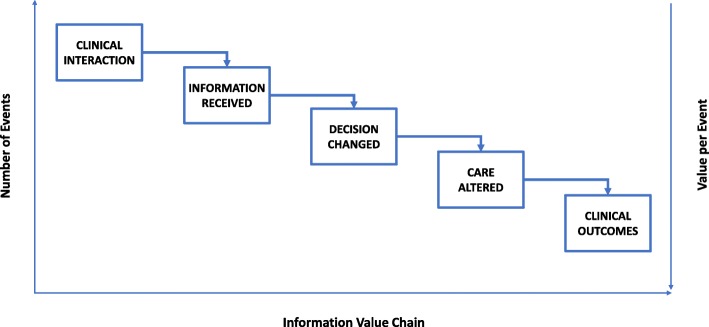
The Information Value Chain. Note: The number of events is typically higher upstream in the information value chain (i.e. closer to clinical interaction), and the events that occur downstream in the information value chain (i.e. closer to clinical outcomes) are more likely to lead to significant clinical changes. *Source: Reproduced with permission from Coiera 2015* [2]

This framing of the role of patient-facing use of telehealth innovations as facilitating patient-provider interactions lends itself to two theoretical approaches to measuring and conceptualising the patient-provider interface – 1) the transaction costs approach, previously used to explore [12] and estimate [13, 14] the costs incurred by patients or providers in terms of the number and duration of steps involved in clinical work flows or consultations; and 2) the value of information (VoI) approach which identifies five steps in an “information value chain” on the path from process to clinical outcomes – see Fig. 1 (i.e. Clinical Interaction → Information Received → Decision Changed → Care Altered → Clinical Outcome) – and has been proposed [2, 3] as a measure to quantitatively estimate value of both medium and message at each step in the process to clinical outcomes continuum. Indeed, a similar approach has been proposed – with the “information value chain” framed as “care cascade” – for separating the effects of innovations on process outcomes from their effects on clinical outcomes [1].

## Transaction costs

The concept of transaction costs captures the consequences of how transactions are governed – i.e. how economic exchange is organised and facilitated – such that the transaction becomes the unit of analysis. Transaction costs reflect the effort, time and monetary costs necessary to complete a given transaction. From the perspective of a consumer seeking to purchase a good (or service), transaction costs are all costs incurred by the consumer that are not transferred to the seller (e.g. the time spent obtaining information on the good or service, and on prices and potential alternatives, legal fees, and the costs of establishing credibility as a buyer) [15]. From the perspective of a producer seeking to sell a good (or service), transaction costs are all costs which the producer would not incur were they selling the good to themself (e.g. time spent waiting as potential buyers examine the good or service, agent and advertising fees, and the cost of establishing credibility as a seller) [15].

Munger argues that in the software-platform revolution that began 20 years ago with the launch of eBay, and continues with the emergence of industry disrupters such as Uber and Airbnb, (and may in time extend to health service delivery), “entrepreneurs have for the first time been able to specialise in selling not more stuff, but in reducing the transaction costs for access to existing stuff.” [16] These platforms reduce transaction costs by providing – information about the identity and location of potential transacting partners; an easy way of paying for services that both parties can trust; and a way of outsourcing reviews or crowdsourcing trust on a provider’s performance of the terms of the contract (including, in some instances, by covering the costs of insurance) [16]. Patient-facing digital health innovations may perform similar functions – e.g. mobile phone apps designed to support self-care in the home or help patients to locate, pay for, and rate laboratory services in their vicinity.

Notably, reduction in transaction costs have implications for the value proposition of patient-facing digital health innovations along two lines – allowing the use of excess capacity and shifting relative transaction costs between stakeholders. First, by reducing transaction costs on the demand or the supply side, patient-facing digital health innovations can maximise the use of currently unused or underused capacity in a system – just as Uber and Airbnb free up time that might otherwise be used to search for transacting partners and make it possible to commercialise a resource (room in a house or car in a garage) that may otherwise be left unused. Patient-facing digital health innovations can do the same in the health system – by reducing transaction costs, they may add value on the supply side by maximising the capacity of a system to deliver more services, and on the demand side by maximising access to health care services that a population may would otherwise not use.

The second implication of reduction in transaction costs of patient-facing digital health innovations is that they can re-distribute relative transaction costs among supply- or demand-side stakeholders. For example, video consultations may reduce transaction costs for patients (e.g. direct and indirect costs of travel, which may be especially high for remote- and rural-dwelling patients as is the case with the children and families we work with in Australia) but at the same time increase transaction costs for the health system (e.g. the costs of installing the videoconferencing equipment, and of re-designing service delivery – as the innovation may impose increased cognitive load on clinicians’ attentional resources [4]). And conversely, a self-monitoring app may increase transaction costs for patients (e.g. due to time spent on monitoring by patients or their family) but may also reduce transaction costs for the health system (e.g. less expense on staff time to monitor patients). Thus, while some stakeholders may see benefit in such innovations, others may not see their value.

The level or extent of transaction costs on any given patient-provider interface is influenced by how care is organised and coordinated, which may be augmented by the use of technology [12–14, 17], (which, in turn, depends on the characteristics of the technology-provided information channel, the context-specific requirements of the information to be transferred between patient and provider, and the relationship between patient and provider [4]). And due to the complex series of transactions involved in service delivery, transaction costs exist, “at every juncture over which information flows… and in all aspects of organizing how care is arranged, by whom (and where) it is provided, and how it is reimbursed.” [18] And the choices made about the organisation of care are reflected in the cost-benefit trade-off between modes of delivery and their governance structures. Because of their comparative efficiency, governance structures with superior transaction costs economising properties tend to displace existing governance structures with worse transaction costs economising properties [19].

Transaction costs provide a framing for measures that may be used to examine the costs or benefits experienced by parties to a transaction. Patient-facing digital health innovations alter the costs (i.e. labour, capital, and technology) required to consummate a transaction, and may free up capacity in a system. Measures that characterise these costs and benefits (quantitative or qualitative) may well be used to assess the value of patient-facing digital health innovations for various stakeholders.

## Value of information

The concept of “Value of Information” is based on decision theory [2, 3, 20], and offers a complementary theoretical lens by which to assess and measure the value of patient-facing health technologies. The Value of Information (VoI) is the difference between the value of embarking on an alternative course of action because of new information (i.e. expected utility of option 1) and the value of persisting with the current course of action or state of affairs (i.e. expected utility of option 2) [2, 3] – i.e.

$$ \mathsf{VoI}=\mathsf{EU}\ \left(\mathsf{Option}\ \mathsf{1}\right)-\mathsf{EU}\ \left(\mathsf{Option}\ \mathsf{2}\right) $$

Note that the “expected utility” (EU) of a given piece of information (e.g. obtained using information technology) or a clinical intervention (e.g. a treatment or a diagnostic procedure) is the probability of improvement or survival with the information or clinical intervention multiplied by a utility value which estimates the cost, pain, and suffering of obtaining the information or undergoing the clinical intervention [2, 3]. For example, if the information transferred between a patient and a provider during a video consultation is the same with in-person consultation, then the difference in EU between the two options – i.e. the VoI – will be found in non-clinical considerations (e.g. transaction costs).

Indeed, VoI is zero whenever new information does not lead to change. And as Coiera argues [2, 3], for a modality of service delivery such as video consultation, the VoI in relation to clinical outcomes will tend towards zero. But the VoI of video consultation in relation to the choice of mode of interaction may be substantial. Other forms of digital health innovation (e.g. a patient-facing continuous patient monitoring system, or a provider-facing decision support system) generate information that is more likely lead to change (in the form of a different or more timely clinical decision). Hence their VoI in relation to clinical outcomes is potentially greater than video consultation which is, after all, simply an alternate form of patient-provider interaction with little or no additional information, compared to a face-to-face interaction. However, this in no way suggests that video consultation is without value; just that its value may not typically be found in relation to clinical outcomes.

Hence, the utility of a telehealth innovation such as video consultation is often in relation to process events that precede and may result in clinical outcomes. Similar to a “care cascade” [1], this series of events exists along what Coiera describes [2, 3] as “a long information value chain” (see Fig. 1) which begins from a user’s (patient or provider) interaction with a source of information and goes through several steps before change manifests in the form of clinical outcome. The first step in Coiera’s five-step information value chain is the *clinical interaction* (where, for example, the VoI of video consultation may be high); the second is *information received* (where the VoI of electronic health records may be high); the third is *decision changed* (where the VoI of decision support systems may be high); the fourth is *care altered* (where VoI of electronic care pathways may be high); and the fifth is *clinical outcome* (where the series of events/steps may culminate in VoI for clinical outcomes) [2, 3].

What the information value chain implies is that “at each step in the chain there is potential for a ‘loss’ [of utility]” [3] such that – 1) not all new information result in a decision being changed; 2) only some decisions result in a change in the process of care, and 3) only some process changes have an impact on clinical outcomes [2, 3]. So, while the VoI of video consultation often tends towards zero in relation to change in clinical outcomes, it may, in fact, be particularly high in relation to the first step in the information value chain – “the clinical interaction”. In the clinical interaction step, the EU of video consultation (for the patient or for the provider), i.e. the utility of providing the same quality of care at distance, may diverge markedly from the EU of the alternative – i.e. the utility (or costs) of in-person interactions. Hence, video consultation can have high VoI when evaluated for its capacity to maximise EU at the interaction stage (rather than its capacity to change clinical outcomes).

Like transaction costs, VoI can serve as a quantitative measure to assess the relative value of patient-facing digital health innovations – i.e. VoI can be computed to compare one telehealth technology with another, and with “usual care” or “current practice”; and especially to measure value at any of the five steps along the information value chain. While transaction costs is only a measure of utility, VoI allows the integration of different measures of utility (including transaction costs) in calculating VoI at each step in the information value chain or care cascade. VoI is a richer, more comprehensive measure of value. Not only can it integrate different measures of utility for a patient or a provider, it can also separate process outcomes from clinical outcomes. And within this framework, transaction costs is a measure which highlights systems level utility – i.e. effects of these innovations on the use of excess capacity, population access and redistributing transaction costs between stakeholders.

In this paper, we identify measures which have so far been used in randomised controlled trials to distinguish the relative value of modalities of service delivery, and we evaluate the extent to which the measures are able to determine transaction costs and VoI along the information value chain.

## Review methods

The studies reviewed included trials that made head-to-head comparison of at least two different modes of delivering the same service. We searched Medline, Embase and Global Health databases via Ovid (from inception to 12 January 2018) using the following terms: #1. [tele*/] AND #2[Patient Outcome Assessment/ OR “Outcome Assessment (Health Care)”/ OR Treatment Outcome/ OR “Outcome and Process Assessment (Health Care)”/ OR Patient Reported Outcome Measures/ OR “Process Assessment (Health Care)”/ OR Patient Satisfaction/ OR patient reported experience measures/]. We limited the search to only randomised controlled trials, published in English and in a population of 0 to 18-year-old children, given the initial motivation for this review. Studies were excluded if the comparator or control was ‘no care’ or ‘usual care’, if the intervention was an add-on to usual care, or if the control and intervention arms had different ‘doses’ of same intervention.

We ensured that the difference between groups in a trial related only to the medium of delivery. When two groups in a trial are able to report the results of remote, home-based tests to providers through different channels (e.g. a mobile phone app versus phone calls), but only one group receives feedback from a provider, the study was deemed as testing the effects of timely feedback rather than the relative effects of the modalities of delivery. To be included, a study comparing an in-person mode of service delivery with an innovation that includes remote patient monitoring and feedback must provide opportunities for contact between scheduled consultations (including unscheduled visits) in order to ensure a comparative ‘dosage’. Otherwise such a trial was deemed as only assessing the effects of regular provider feedback. For trials with more than two intervention arms, we only included the arms in which groups received essentially the same intervention.

Quality assessment of included trials was conducted using the revised Cochrane risk-of-bias tool for randomised trials [21] – along its five domains: (1) bias arising from the randomization process; (2) bias due to deviations from intended interventions; (3) bias due to missing outcome data; (4) bias in measurement of the outcome; and (5) bias in selection of the reported result – based on the primary outcome measure in each study. Most of the trials are of low risk, four studies have some concerns, and none is of high risk (see Table 1). No study was excluded due to concerns about risk of bias.

**Table 1:** The four categories of randomised controlled trials of telehealth interventions for children included in the review and the measures used to assess their value

Group	Study	Modality	Service	Risk of Bias	Outcome	Satisfaction	Cost
1	Nelson et al. 2011 [22]	Text message versus telephone message	Reminder of dental care visit	Low Risk	Yes^1DC^	No	No
1	Goldman et al. 2004 [23]	Email versus telephone call	Reminder of post-emergency care visit	Low Risk	Yes^1IR^	Yes^3D^	No
1	Bigna et al. 2014 [24]	Text message versus telephone call	Reminder of HIV care follow-up visit	Low Risk	Yes^2DC^	No	Yes^1S^
1	Szilagyi et al. 2013 [25]	Letter versus telephone message	Reminder of immunisation and preventive visit	Low Risk	Yes^2CA^	No	Yes^1S^
1	Vivier et al. 2000 [26]	Letter versus telephone call	Reminder of immunisation visit	Low Risk	Yes^2CA^	No	No
1	Franzini et al. 2000 [27]	Postcard versus telephone message	Reminder of immunisation visit	Low Risk	Yes^2CA^	No	Yes^1S^
1	Dini et al. 2000 [28]	Letter versus telephone message	Reminder of immunisation visit	Low Risk	Yes^2CA^	Yes^3D^	Yes^3S^
1	Lieu et al. 1998 [29]	Letter versus telephone message	Reminder of immunisation visit	Low Risk	Yes^2CA^	Yes^3D^	Yes^1S^
2	Looman et al. 2015 [30]	Videoconferencing versus telephone call	Care coordination in complex multimorbidity	Low Risk	No	Yes^2D^	No
2	McCrossan et al. 2012 [31]	Videoconferencing versus telephone call	Home support in congenital heart disease	Low Risk	No	Yes^1S,1D^	Yes^1S^
2	Morgan et al. 2008 [32]	Videoconferencing versus telephone call	Home support in congenital heart disease	Low Risk	Yes^1CO^	Yes^1S,1D^	No
2	O’Shea et al. 2007 [33]	Telephone call versus in-person	Post-discharge follow-up in chronic lung disease	Low Risk	Yes^2CO^	No	Yes^2S^
2	Cadario et al. 2007 [34]	Internet versus in-person	Blood glucose monitoring in type 1 diabetes	Some Concerns	Yes^1CO^	No	Yes^1S^
2	Carlsen et al. 2017 [35]	Internet versus in-person	Monitoring in Inflammatory Bowel Disease	Low Risk	Yes^2CO^	Yes^3D^	Yes^1S,1D^
2	Akobeng et al. 2015 [36]	Telephone call versus in-person	Monitoring in Inflammatory Bowel Disease	Some Concerns	Yes^2CO^	Yes^2D^	Yes^1S^
2	Tsafack et al. 2015 [37]	Telephone beep versus in-person	Post-immunisation monitoring of adverse events	Low Risk	Yes^1CI^	No	No
3	Catenacci et al. 2014 [38]	Internet versus printed material	Intervention to reduce sedentary behaviour	Low Risk	Yes^2CO^	Yes^1D^	No
3	Davis et al. 2016 [39]	Videoconferencing versus telephone call	Behavioural group intervention for obesity	Low Risk	Yes^2CO^	Yes^2D^	No
3	Plonka et al. 2013 [40]	Telephone call versus in-person	Intervention to prevent dental caries	Low Risk	Yes^1CO^	No	No
3	Chan et al. 2007 [41]	Internet versus in-person	Education in asthma	Low Risk	Yes^1CA^	Yes^2D^	Yes^2S,1D^
3	Patten et al. 2006 [42]	Internet versus in-person	Counselling for smoking cessation	Low Risk	Yes^2CO^	Yes^1D^	No
3	Oda et al. 1995 [43]	Telephone versus in-person	Education to receive preventive services	Low Risk	Yes^2CA^	No	No
3	Ruble et al. 2013 [44]	Internet versus in-person	Teacher coaching on autism	Low Risk	Yes^2CO^	Yes^2S,2D^	No
3	Duke et al. 2016 [45]	Videoconferencing versus in-person	Intervention on family support in diabetes	Low Risk	Yes^2CO^	No	No
4	Turner et al. 2014 [46]	Telephone versus in-person	CBT for obsessive compulsive disorder	Low Risk	Yes^2CO^	Yes^1D^	No
4	Comer et al. 2017 [47]	Videoconferencing versus in-person	CBT for obsessive compulsive disorder	Low Risk	Yes^2CO^	Yes^2S,2D^	No
4	Levy et al. 2017 [48]	Telephone versus in-person	CBT for functional abdominal pain	Low Risk	Yes^2CO^	Yes^2D^	Yes^2S,2D^
4	Himle et al. 2012 [49]	Videoconferencing versus in-person	Behaviour therapy for tic disorder	Low Risk	Yes^2CO^	Yes^2D^	No
4	Grogan-Johnson et al. 2012 [50]	Videoconferencing versus in-person	Speech therapy	Some Concerns	Yes^2CO^	Yes^3S,3D^	No
4	Nelson et al. 2003 [51]	Videoconferencing versus in-person	CBT for depression	Low Risk	Yes^1CO^	Yes^2D^	No
4	Kopycka-Kedzierawski et al. 2013 [52]	Videoconferencing versus in-person	Assessing dental caries	Low Risk	Yes^2CA^	Yes^3S,3D^	No
4	McConnochie et al. 2016 [53]	Videoconferencing versus in-person	Evaluation and treatment of acute illnesses	Some Concerns	Yes^2CA^	No	No

Note: Internet modality refers to interactive websites/apps; *CBT* Cognitive Behaviour Therapy and Superscript 1 = significant, 2 = not significant, 3 = no head-to-head comparison; *D* demand side, *S* supply side, *CI* Clinical Interaction, *IR* Information Received, *DC* Decision Changed, *CA* Care Altered, *CO* Clinical Outcome

## Review findings

**Fig. 2:**
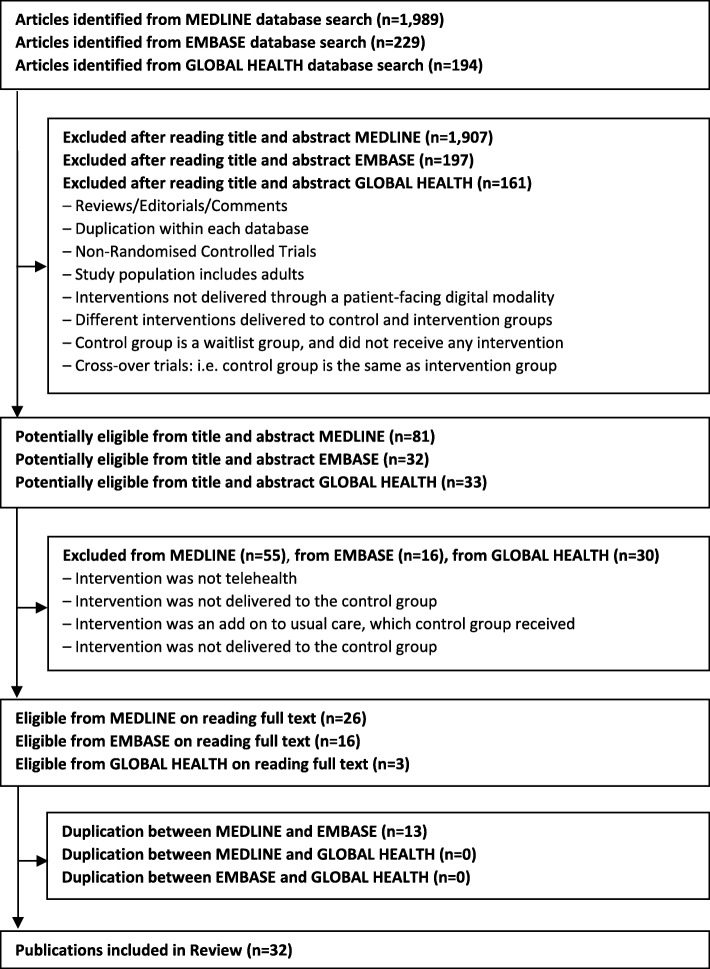
Flow Diagram of the Review of Measures Used to Assess the Value of Patient-Facing Telehealth

The initial search returned 2412 publications – 1989 from Medline, 229 from Embase, and 194 from Global Health. After reading the title and abstracts, a total of 146 were selected – 81 from Medline, 32 from Embase, and 33 from Global Health. And after reading the full text of each, and eliminating duplications, we identified 42 publications. Further examination of the full text resulted in a total of 32 publications included in the review (see Fig. 2). The information from the articles was exported into Excel spreadsheets, and the following data was extracted: title of article, first author, year of publication, location of the study, income group of study participants (high versus low income), geographical setting (rural versus urban), modalities of delivery being compared, the potential rationale for considering a new modality of service delivery, the relevant research question being addressed, measures used in evaluation and explanation of evaluation outcomes.

Two of the 32 trials were conducted in a low- or middle-income country; both in Cameroon. Of those in high-income countries, 22 were in the United States of America, four in the United Kingdom, and one in each of Australia, Canada, Denmark and Italy. Only four trials were exclusively of low-income populations; 25 were in urban, four in a mix of both urban and rural, and three in rural locations. Through an iterative process, we identified three categories of measures used to evaluate modalities in the trials: outcome (process and clinical), satisfaction and cost (Table 1). We then analysed each measure based on whether there was statistically significant difference between mediums. For the outcome measures, we identified the point along the information value chain at which they were measured; and for the satisfaction and costs measures, we identified whether they were assessed on the demand-side (e.g. a patient or their family) or the supply-side (e.g. provider, payer or health system). Notably, none of the trials reported on measures of system capacity or population access (e.g. change in number of health workers per patient, wait lists or level of population coverage).

We grouped the trials into four categories based on the service being delivered through a patient-facing telehealth technology: 1. information for initial or a follow-up patient-provider contact; 2. post-discharge patient monitoring; 3. behaviour change and educational interventions; and 4. treatment and diagnosis. There were, incidentally, eight trials in each category.

### 1. Facilitating initial or follow-up patient-provider contact

#### Outcome

While all eight trials measured outcome, only two showed significant difference between modalities – in one of the trials, this difference in outcome (“decision changed”) was attributed to parents’ preference for and being already accustomed to the more effective modality (i.e. telephone message) compared to text message reminder [22]; in the other trial in which outcome was measured as “information received” the less effective modality (i.e. email reminder) was still relatively new among patients (compared to telephone call reminder) in 2003 when the trial was conducted [23].

#### Satisfaction

Only three of the eight trials measured satisfaction with the modalities and they measured only demand-side satisfaction; but did not include a head-to-head comparison between modalities. In one, satisfaction was only assessed in one group, with two closed questions (one of which was framed positively) and positive options listed first, resulting in overwhelming positive responses [28]. In another in which satisfaction was also measured in one group (the telephone group), with 12 closed questions administered to parents, again, most parents responded positively [29]. In yet another, satisfaction was not only assessed in one group, but just among the sub-set of that group who did not respond to the reminder, to understand why they did not respond [23].

#### Cost

Four of the eight trials had formal economic evaluations (all from a supply-side perspective), with the tendency that simpler modalities were more cost-effective; e.g. in text message versus telephone call, text message was more cost-effective – although telephone calls became more cost-effective when the direct cost of the telephone calls (i.e. fees paid to telephone companies, potentially borne by a third party) was excluded [24]; and in letter versus telephone message, letter was more cost-effective – although the cost-effectiveness of letters may have been limited as this trial was conducted among low-income households with low literacy [25]; and in postcard versus automated telephone message, postcard was more cost effective – although telephone message was more cost-effective at scale (i.e. in large practices) [27]. However, in one trial of letter versus telephone message, telephone message was more cost-effective – and even more so under the assumption that the intervention would not require additional computer hardware costs and that the software for the automated telephone message would not require a programmer [29].

### 2. Facilitating ongoing post-discharge patient monitoring

#### Outcome

Six of the eight trials reported on outcomes, and half of them demonstrated significant difference between modalities; differences which are due to mechanisms afforded by the inherent properties of a modality – e.g. videoconferencing was significantly more effective than telephone call in reducing anxiety among parents of children with congenital heart disease (i.e. “clinical outcome”) [32]; internet-based reporting of and feedback on glucometer reading was more effective with (adolescent) diabetes patients than in-person appointments in achieving reduction in glycated haemoglobin (i.e. “clinical outcome”), probably because the ease of communication over the internet allowed the feedback to the internet group to additionally include social and diet issues of peculiar concern to adolescents [34]; and the opportunity to report adverse events following immunisation through a telephone beep led to significantly more events being reported (i.e. “clinical interaction”) than in-person because the modality removed two potential barriers – the cost of travel to the health facility to report, and the cost of making the telephone call, as they only had to beep, and the health facility called back in response [37].

#### Satisfaction

Five of the eight trials assessed indicators of satisfaction; two of which (comparing videoconferencing versus telephone monitoring of children with congenital heart disease) did so on both demand- and supply-sides [31, 32]. On the supply side, these two trials assessed how the presence or lack of visual assessment influenced clinician decision making – clinicians were more confident making decisions and addressing parents’ concerns with videoconferencing [31, 32]; and on the demand-side, parent satisfaction was assessed in both trials using a Likert scale – parents were more satisfied with videoconferencing [31, 32]. The three trials that only examined demand-side satisfaction either found no significant differences (videoconferencing versus telephone care coordination for children with medical complexity [30], and telephone versus in-person monitoring of Inflammatory Bowel Disease [36]) or had no head-to-head comparison between the modalities (internet versus in-person monitoring of Inflammatory Bowel Disease [35]) as satisfaction was assessed only in the internet arm.

#### Cost

Of the five trials that empirically measured costs or indicators of costs, none measured costs on the demand-side; only costs incurred (or saved) on the supply-side in terms of consultation time, contact with the health system, and hospitalisation. In two trials, consultation time was significantly reduced by modalities other than in-person (telephone call [36] and internet-based application [34]). Of the four trials in which hospitalisation rate was measured, one found significant reduction – it was attributed to clinicians’ greater confidence in decision-making with visual assessment [31]; and three found no significant difference comparing two modalities (telephone [33, 36] and internet [35]) with in-person delivery. Of the three trials that compared episodes of contact with the health system, significant reduction was found only in the total number of contacts or visits [31, 35] and in unscheduled visits [31] – but not in scheduled contacts [34]. Only one out of these five trials (internet-based versus in-person monitoring) assessed demand side costs – and it found significant reductions in disease-related school absence [35].

### 3. Facilitating the delivery of educational and behavioural interventions

#### Outcome

Outcome was assessed in all eight trials, but only in two of them was there a significant difference observed between modalities: 1. telephone versus in-person dental education in which more children in the telephone group had *Streptococcus mutans* in their teeth as compared to those who received home visits which allowed dental educators to personally instruct mothers in oral hygiene techniques, resulting in more effective toothbrushing i.e. a “clinical outcome” which is indeed a direct result of “care altered”) [40]; and 2. internet versus in-person asthma education and case management – which demonstrated greater improvement in knowledge, practice and adherence to prevention activities in the internet group (i.e. care altered) [41].

#### Satisfaction

Five trials measured satisfaction, all of them using indirect measures such as adherence or fidelity – only one measured satisfaction directly [39]; and in addition only one measured satisfaction on the supply-side [44]. The only trial (videoconferencing versus telephone) in which satisfaction was measured directly (on a 10-point scale), found no significant difference – even though parents in the telephone group desire ‘at least one face-to-face visit to make the program more meaningful’ – and neither were significant differences observed for indirect measures such as attendance or sessions completed [39]. Only two trials with indirect measures of satisfaction showed significant differences in participation or attendance, in favour of internet delivery (versus printed material – suggesting that the modality was more engaging [38]; and versus in-person – but attendance in the internet group subsequently dropped [42]). The two trials that measured satisfaction indirectly and demonstrated no difference were also of internet versus in-person delivery. One of them measured satisfaction in terms of participation rate [41]. And the other one measured satisfaction in terms fidelity (this was the only trial in this category to measure satisfaction on both demand- and supply-sides) – coaches rated the fidelity of teachers in implementing teaching plans using Likert scale (supply-side), teachers rated the fidelity of coaches to the coaching protocol using 16-item scale (supply-side) and patients rated the fidelity of coaches to the coaching protocol using a 25-item scale (demand-side) [44].

### Cost

Only one trial (comparing internet versus in-person delivery) assessed costs; and it was on both demand- and supply-sides [41]. On the supply-side, the number of total visits (i.e. both scheduled and unscheduled) was significantly greater for the in-person compared to the internet group, but there was no significant difference in unscheduled emergency visits and hospitalisations. On the demand-side, there was significant difference in the use of time – parents in the internet group spent half as much time (away from work and in travelling to access care) as those in the in-person group.

### 4. Facilitating the delivery of treatment and diagnostic services

#### Outcome

All eight trials assessed for efficacy, but only one showed greater efficacy of one modality (videoconferencing over in-person delivery); in this case in relation to a “clinical outcome”(the rate of reduction in the symptoms of depression), attributed to the novelty of videoconferencing in the population [51]. Of the seven remaining trials (i.e. those without such significant difference), five were videoconferencing versus in-person delivery (three in relation to a “clinical outcome” and two in relation to “care altered”), and two were telephone versus in-person delivery (both of them in relation to a “clinical outcome”).

#### Satisfaction

Of the eight trials, seven measured satisfaction; but in only one trial (telephone versus in-person) was there significant difference between modalities (more patients were “very happy” with being allocated to the telephone group), although no significant difference was reported by patients and parents in satisfaction (using a questionnaire), treatment credibility (using a treatment credibility and expectancy scale) or treatment alliance (using a measure of therapeutic alliance) [46]. And four trials showed no significant difference in either direct or indirect measures of demand-side satisfaction: three of videoconferencing [47, 49, 51] and one of telephone [48] versus in-person delivery (with direct measures of satisfaction such as child- and parent-rated acceptability, therapeutic alliance, satisfaction and session tardiness; and indirect measures of satisfaction such as retention and attendance rates). In two trials (videoconferencing versus in-person) satisfaction was only measured in the videoconferencing group, albeit on both the demand- and supply-sides [50, 52]. In only one trial (videoconferencing versus in-person), was there a head-to-head comparison of supply-side satisfaction (therapist-reported treatment alliance), but without significant difference [47].

#### Cost

Only one trial measured costs, doing so on both the demand- and supply-sides (telephone versus in-person) on the demand-side – use of time (number of missed school days) and number of contacts with the health system, without significant difference between modalities [48].

## Discussion

There is wide variation in the literature on how to measure the value of patient-facing digital health innovations such as telehealth. However, we identified three groups into which the measures may be categorised: outcome (process and clinical), satisfaction and cost. Measures of outcome do vary widely, as these innovations are put to different clinical uses. But it is possible, as we argued earlier in this paper, to have a common metric (i.e. mathematical combination of measures) by which to evaluate their relative value – by using VoI and incorporating transaction costs as a measure of utility. More trials assessed outcome in terms of clinical outcomes, compared to all process outcomes put together – 17 trials compared to 13 trials. And of the trials that assessed satisfaction, most did so on the demand side, while most of the trials that assessed costs did so on the supply side. Expectedly, only a minority (eight out of 30) of trials in which outcome was measured showed significant difference between modalities – one showed the superiority of in-person delivery [40], while four demonstrated the superiority of patient-facing telehealth innovation over in-person delivery [34, 37, 41, 51], and three demonstrated the superiority of one patient-facing telehealth innovation over another patient-facing telehealth innovation [22, 23, 32].

Notably, demonstrated differences in outcome (process or clinical) between modalities of delivery often appeared to be contextual; influenced (as judged by the researchers who conducted the trials) by various aspects of context. In other words, the comparative effectiveness of different modalities of delivery would probably vary depending on the population/location, the time/historical period or the duration of the trial. Some differences in outcome were attributed to: 1) novelty – leading to the superior effect on “clinical outcome” of video consultation compared to in-person service [51], and the superior effect on “information received” of synchronous messaging (i.e. telephone call) compared to asynchronous messaging (i.e. email) [23]; 2) existing preference for one modality over another – in this case, between two asynchronous messaging modalities, with voice messages being superior to text messages in relation to “decision changed” due to previous engagement with the voice of clinic reception staff [22]; and 3) the extent to which a digital health innovation reduced the transaction costs of accessing services – in a trial in which a telephone beep was compared to in-person reporting of adverse events significantly increased the incidence of reporting – i.e. “information received” [37].

In other trials, the reason why one modality of delivery was superior (in terms of a process or clinical outcome) was explained (or is indeed explainable) by the higher level of information or interaction afforded by the superior modality which eventuated, down the information value chain, in improved “care process” or “clinical outcome” [32, 34, 40, 41]. In all, findings on differences in the relative efficacy of alterative modalities of service delivery are in keeping with theoretical predictions: [4] 1) that although patients (and/or their family) are rational agents, they make decisions under conditions of scarcity – of time, financial and attentional resources; and 2) that the extent to which patients and providers are willing to deploy or respond to in-person or patient-facing digital interactions, is based on how they assess the cost and benefit of past interactions and current goals. Thus, messages that are easy to understand, that are shared between patient and provider with existing relationships, through a familiar medium, and are neither disruptive nor a burden on attentional resources (e.g. by being synchronous or asynchronous; or by being accessible at one’s convenience through the internet or requiring in-person visits) are more likely to be effective along the information value chain [2, 4].

With regard to the satisfaction category, of the 14 trials with head-to-head comparison of demand-side satisfaction, only five demonstrated significant difference – three of which showed superiority of a digital innovation over in-person delivery [38, 42, 46], and two showed superiority of one digital innovation over another [31, 32]. On the other hand, only three trials included head-to-head comparison of supply-side satisfaction, of which two showed superiority of one modality of delivery over another (video over audio) with the superiority of video delivery explained by the higher level of information available to a clinician through video. Notably, in one trial, this translated into a significant reduction in supply-side costs (i.e. transaction costs of delivering services) [31] – because clinicians made fewer unnecessary referrals in the video group – thus, demonstrating a link between costs and supply-side satisfaction. Of the 11 trials with head-to-head comparison of supply-side costs, eight demonstrated superiority of one modality of delivery over another – three of a digital innovation over in-person delivery [34–36], and five of one non-in-person delivery over another [24, 25, 27, 29, 31]. On the other hand, only three trials had head-to-head comparison of demand-side costs (i.e. transaction costs of accessing services), of which two showed superiority of one modality (digital) over another (in-person) [35, 41].

Essentially, each of the three categories of measures relate to the others and they can co-determine one another. Of all the categories of measures, the cost category was most discriminating between modalities of delivery in terms of showing one to be superior over another (two out of three trials on the demand side and eight out of 11 trials on the supply side) – compared to satisfaction (five out of 14 on the demand side compared to two out of three on the supply side) and outcome (eight out of 30 trials). However, the costs were analysed at the individual level. While outcome and satisfaction measure value at the individual level and may be peculiar to individuals, costs have a dimension of scale. In three of the trials included in this review, it was suggested that cost-effectiveness may be altered significantly depending on factors that are sensitive to the scale of implementation [24, 27, 29]. Efforts to scale up patient-facing digital health innovations must consider how costs (especially supply-side costs) change with scale, and how reduced transaction costs can lead to improved access and service uptake, which in turn may alter the cost-effectiveness at scale. Implementation at scale may also influence supply-side satisfaction (as clinicians are required to deliver greater volume of services) and population health (as more people are able to access services).

Notably, the studies that demonstrated the supply-side cost saving effect of patient-facing digital health technologies did so primarily by their effects on the total number of visits a patient makes or needs to make to a health facility [31, 35, 41] – not on the number of unscheduled visits (to a clinic or to an emergency department or for hospitalisation) [35, 41]. This may be because the number of unscheduled visits is more attributable to the effectiveness of the message (i.e. intervention), and to context (e.g. distance to health facility) than the medium or modality of delivery itself. Nonetheless, the impact of patient-facing digital health innovations on the transaction costs incurred by patients or providers is important in determining their value – the transaction costs of accessing services for the individual patients (on the demand side), and the transaction costs of delivering services by the health system (on the supply side). By reducing the direct and indirect costs of accessing services, the effects of these innovations on reducing the transaction costs of accessing services (i.e. increasing population access to services) are particularly important for low-income and rural or remote dwelling families who tend to access services at a distance. Likewise, their effects on reducing the transaction costs of providing services (i.e. helping to achieve economies of scale and to maximise excess capacity within a system) are important for health systems globally, and may be more important to the future.

Future research on patient-facing digital health innovations should assess both supply and demand-side transaction costs. However, reduction in transaction costs is not an end in itself; and the mere measurement of transaction costs is not sufficient. The evaluations of these innovations should also measure how and to what extent they influence population access to services, economies of scale and the creation and use of excess capacity. Likewise, satisfaction needs to be measured on both the demand and supply sides – among the trials included in this paper, satisfaction was often measured only on the demand side. In addition, transaction costs may be incorporated into measures used to assess supply- or demand-side satisfaction by different stakeholders – because the extent of change in transaction costs may represent a surrogate measure of satisfaction [1]. Returning to the definition of Value of Information (VoI) – i.e. as the difference in expected utility (EU) between two options of delivering a piece of information or a clinical intervention [2, 3] – we argue that efforts to determine the value of patient-facing digital health innovations should include measures which estimate (on both the supply and demand sides) the extent of change in transaction costs and the level of satisfaction associated with obtaining information or service through the alternatives under consideration.

However, the set of variables included in the economic and decision-theoretic models to assess the EU (and by extension, the VoI) while comparing a patient-facing digital health innovations with an alternative modality of service delivery should explicitly consider their long-term effects on both population access to services and system capacity to deliver services. These innovations can radically alter the extent of capacity within a system and the pattern of future investments within health sector. The models should also incorporate multiple dimensions of the attributes of each alternative that may influence choice on the demand or supply sides. The measures of satisfaction in the trials included in this review were unidimensional. Indeed, preference may be a more robust approach to assessing satisfaction – hence a method such as discrete choice experiments may help to better assess preferences [54, 55] – and inform more comprehensive estimates of utility. Such an approach may also benefit from incorporating different levels of demand or supply side transaction costs into the range of choices presented to the stakeholders while assessing preference – to understand which innovations stakeholders are willing to adopt, and also better assess the potential for spread, scale up and sustainability. Estimates of utility should also incorporate willingness to pay studies [56, 57] – which may also define what is being paid for in terms of reduction in transaction costs.

In this review, we did not discriminate based on the quality of individual trials. This was because our aim was primarily to assess existing practice in the choice of the measures which are used to assess the value of patient-facing digital health innovations. For example, we included pilot and feasibility trials as we were equally interested in the choices of measures of value made by the researchers in those trials. However, it was often unclear what informed the choices of authors as to why value was measured in some of the three categories and not others; or why value was measured on the supply or demand side. The reason could range from custom (i.e. tradition in a field), a theorised/expected high likelihood of detecting or not detecting effect, or a reflection of systematic bias – i.e. post-hoc selection of measures to report. Trials conducted in the public sector (trials rarely occur in private settings) may not have measured excess service delivery capacity because there is often no excess capacity in saturated public systems where innovations only serve to improve population access. But if our aim is indeed to understand the potential for adoption and scale up, and to compare among innovations, then trials need to assess value using consistent measures, on both the supply and demand sides. Reporting guidelines for trials of patient-facing innovations should require that omissions of measures should be pre-specified and adequately theorised.

## Conclusion

In summary, this review shows that trials of patient-facing digital health innovations have used three categories of measure to evaluate the relative value of a medium of delivery: outcome (process or clinical), satisfaction and cost. Even though these innovations tend to confer value by their effects on process outcome, satisfaction, cost (i.e. transaction costs), and other related non-clinical outcome measures, these measures are often either not assessed or inconsistently assessed. We argue – 1) that efforts (of policy makers, implementers, researchers, innovators, and funders) to determine their relative value (on the supply and/or demand side) should incorporate all categories of measure into a metric that is rich and comprehensive enough to capture all the potential dimensions of value; 2) that the Value of Information (VoI) is one such metric – calculated as the difference between the expected utility (EU) of two alternative options; and 3) that EU should be estimated by multiplying the probability of achieving not only a clinical outcome, but also process outcomes (depending on the innovation or outcome under consideration) with measures of utility that include measures such as transaction costs, satisfaction, population access, system capacity et cetera. Indeed, VoI may well be the answer the question that led to this review: i.e. *by what measure should a policy maker choose between two mediums that deliver the same or similar message or service?*

## Data Availability

The data supporting the findings detailed in this review is available on request from the corresponding author.

## Abbreviations

EU: Expected Utility
VoI: Value of Information
